# Amplified and Homozygously Deleted Genes in Glioblastoma: Impact on Gene Expression Levels

**DOI:** 10.1371/journal.pone.0046088

**Published:** 2012-09-28

**Authors:** Inês Crespo, Hermínio Tão, Ana Belen Nieto, Olinda Rebelo, Patrícia Domingues, Ana Luísa Vital, Maria del Carmen Patino, Marcos Barbosa, Maria Celeste Lopes, Catarina Resende Oliveira, Alberto Orfao, María Dolores Tabernero

**Affiliations:** 1 Centre for Neuroscience and Cell Biology, University of Coimbra, Coimbra, Portugal; 2 Faculty of Pharmacy, University of Coimbra, Coimbra, Portugal; 3 Neurosurgery Service, University Hospital of Coimbra, Coimbra, Portugal; 4 Centre for Cancer Research (CIC IBMCC-CSIC/USAL), Department of Medicine, University of Salamanca, Salamanca, Spain; 5 Neuropathology Laboratory, Neurology Service, University Hospital of Coimbra, Coimbra, Portugal; 6 Department of Statistics, University of Salamanca, Salamanca, Spain; 7 Faculty of Medicine, University of Coimbra, Coimbra, Portugal; 8 Instituto de Estudios de Ciencias de la Salud de Castilla y León (IECSCYL), Soria, Spain; 9 Instituto Biosanitario de Salamanca (IBSAL) - Research Unit of the University Hospital of Salamanca, Salamanca, Spain; Harvard Medical School, United States of America

## Abstract

**Background:**

Glioblastoma multiforme (GBM) displays multiple amplicons and homozygous deletions that involve relevant pathogenic genes and other genes whose role remains unknown.

**Methodology:**

Single-nucleotide polymorphism (SNP)-arrays were used to determine the frequency of recurrent amplicons and homozygous deletions in GBM (n = 46), and to evaluate the impact of copy number alterations (CNA) on mRNA levels of the genes involved.

**Principal Findings:**

Recurrent amplicons were detected for chromosomes 7 (50%), 12 (22%), 1 (11%), 4 (9%), 11 (4%), and 17 (4%), whereas homozygous deletions involved chromosomes 9p21 (52%) and 10q (22%). Most genes that displayed a high correlation between DNA CNA and mRNA levels were coded in the amplified chromosomes. For some amplicons the impact of DNA CNA on mRNA expression was restricted to a single gene (e.g., *EGFR* at 7p11.2), while for others it involved multiple genes (e.g., 11 and 5 genes at 12q14.1–q15 and 4q12, respectively). Despite homozygous del(9p21) and del(10q23.31) included multiple genes, association between these DNA CNA and RNA expression was restricted to the *MTAP* gene.

**Conclusions:**

Overall, our results showed a high frequency of amplicons and homozygous deletions in GBM with variable impact on the expression of the genes involved, and they contributed to the identification of other potentially relevant genes.

## Introduction

Current knowledge about the pathogenesis of glioblastoma multiforme (GBM) has unveiled the many genetic and molecular alterations of its tumoral genome [1], [2], [3], [4], [5], [6]. Such alterations often result in deregulation of one or multiple oncogenic pathways, leading to increased cell proliferation and tumor growth [5], [6], [7], [8], [9]. Among other alterations, changes in the dosage, sequence or structure of cancer-related genes have been recurrently identified, mostly in cases that carry DNA copy number (CN) alterations of those chromosomal regions where such genes are coded [2], [3]. In recent years, several studies have highlighted the relevance of recurrent gains of chromosome 7p, together with 10q and 9p21.3 losses, and other less frequent chromosomal alterations [10], [11], [12], [13]. Such numerical chromosomal changes frequently reflect amplification/mutation of the *EGFR* gene (a tyrosine kinase receptor coded at 7p11.2) and loss of the *PTEN* (10q23.31) and *CDKN2A* (9p21.3) tumor suppressor genes [3], [13], [14], [15], [16]. In addition, the *MDM4* (1q32.1), *PDGFRA* (4q12), *CDK4* (12q14.1), and *MDM2* (12q15) genes are also frequently amplified in GBM, whereas inactivation of the *NF1* (17q11.2), *TP53* (17p13.1) and *IDH1/2* (2q34/15q26.1) genes through deletion and/or mutation is also commonly observed [8], [17], [18], [19], [20], [21].

Current availability of large-scale whole genome and gene expression profiling (GEP)-arrays provides an invaluable tool for detailed delineation of those genes involved in recurrent CN alterations, and rapid assessment of their impact on the expression levels of the involved genes. Gain-of-function mutations and both silencing and other type of mutations, may lead to oncogene activation and loss of function of tumor suppressor genes, respectively. Similarly, gene amplification and homozygous deletion may also contribute to the development of the tumoral phenotype. Consequently, detailed analysis of the recurrently involved genes in amplicons and homozygous deletions is a particularly attractive approach for identification of relevant targeted genes. Such analysis would be particularly informative when combined with parallel assessment of the impact of these alterations on the levels of expression of the candidate genes, in paired DNA and mRNA samples.

Previous array-based studies in which CN alterations are related to GEP have contributed to the identification of cancer associated genes relevant to GBM (e.g. *MYCN*, *PIK3CA*, *CCND2*, *KRAS*, *CHD5*, *CXCL12*, *PTER*, *LRRN6C, ERRFI1* and *TACC3*, and at the same time they have confirmed the pathogenic role of other known genes (e.g. *EGFR*) [8], [22], [23], [24], [25], [26]. More recently, The Cancer Genome Atlas (TCGA) research network [8] has further reported occurrence of homozygous deletions of the *NF1* and *PARK2* genes, and amplification of the *AKT3* gene –less frequently also of the *FGFR2* and *IRS2* genes–, in a series of 206 GBM. Other candidate genes for which different mechanisms of alteration (e.g. epigenetic silencing) have been reported in GBM, include the *RBBP5* gene (a member of the *RB* pathway) amplified at 1q32 [23], the *CXCL12* (*CXCR4* ligand involved in chemoattraction and tumor invasion) and the *HK1*(a member of the hexokinase family, known to regulate apoptotic pathways) genes [25]. Nevertheless, high-resolution analyses of somatic CN alterations from a large series of cancer specimens (n = 3,131) including GBM, identified 158 regions which are frequently altered across multiple cancer types, many of which could not be explained by previously known cancer-associated genes [27]. Despite all the above, currently there is limited information [8], [23], [24], [25] about the impact of DNA CN alterations on gene mRNA expression levels in GBM..

In order to identify potentially targeted relevant genes, here we investigated the impact of CN alterations on the expression profile of those genes recurrently involved in amplicons and homozygous deletions in GBM. Our results show that most genes for which a high correlation was observed between CN alterations and gene expression levels, are coded in those chromosomal regions for which amplicons were detected, pointing out the potential role of several genes coded in chromosomes 12q14(e.g. *RAP1B*, *MDM2* and*GRIP1*), 4q12 (e.g. *TMEM165*, *FIP1L1* and *EXOC1*), in addition to the *EGFR* gene, in GBM. Conversely, the *MTAP* gene coded in chromosome 9p21 was the only gene involved in homozygous deletions whose expression levels showed a significant correlation with the CN status.

## Materials and Methods

### Patients and samples

A total of 46 caucasian patients diagnosed with primary GBM in the absence of other known genetic disorders (except for a case –G23– who had a von Willebrandt disease) who were admitted to the University Hospital of Coimbra (Coimbra, Portugal), were included in this study; 21 were males and 25 females with a mean age of 62±13 years (range: 30 to 84 years) (Table 1).

**Table 1:** Clinical characteristics of GBM patients (n = 46) included in this study with information about the type of SNP-arrays used in each case to investigate DNA CN alterations.

Case ID	Age	Gender	Karnofsky Index (%)	Tumor localization	Surgical removal	No. of relapses	Survival after surgery (months)	SNP-array analyzed
G6*	70	Female	80	Temporal	ST	1	19	500K
G8*	67	Female	90	Deep	ST	0	9	500K
G10*	35	Female	80	Temporal	ST	0	15	500K
G12*	74	Male	70	Temporal	ST	0	1	500K
G13*	39	Female	90	Frontal	ST	1	21	500K
G14*	69	Female	70	Frontal	ST	0	0	500K
G15*	79	Male	80	Parietal	T	0	5	500K
G17*	30	Female	80	Temporal	ST	3	67	500K
G23*	50	Female	80	Frontal	ST	0	14	500K
G30*	71	Female	60	Temporal	ST	0	9	500K
G34*	69	Male	80	Temporal	ST	0	5	500K
G35*	50	Female	50	Frontal	ST	0	2	500K
G37*	70	Male	80	Temporal	T	1	32	500K
G39*	70	Female	70	Frontal	ST	1	18	500K
G40*	45	Female	80	Frontal	ST	1	15	500K
G42*	67	Male	80	Temporal	ST	0	2	500K
G44*	48	Male	80	Frontal	ST	0	22	500K
G45*	76	Female	60	Temporal	ST	0	10	500K
G46*	62	Male	60	Frontal	ST	0	3	500K
G50*	84	Male	70	Temporal	ST	0	11	500K
G51*	60	Male	60	Temporal	ST	0	2	500K
G52*	56	Male	90	Frontal	ST	0	21	500K
G53*	74	Male	60	Frontal	T	0	29	500K
G55	54	Female	80	Frontal	ST	1	17	500K
G65	69	Female	60	Parietal	ST	0	1	6.0
G66	60	Male	80	Occipital	T	0	14	6.0
G67	68	Female	80	Parietal	ST	0	35	6.0
G68	72	Male	70	Insular	T	0	26	6.0
G70	56	Female	80	Occipital	ST	0	21	6.0
G71	66	Female	60	Parietal	ST	0	10	6.0
G72	77	Female	70	Temporal	ST	0	1	6.0
G73	78	Female	60	Parietal	ST	0	4	6.0
G79	71	Female	60	Occipital	ST	0	6	6.0
G80	43	Male	80	Frontal	T	1	18	6.0
G81	62	Female	70	Frontal	ST	0	13	6.0
G82	78	Male	70	Frontal	ST	0	2	6.0
G83	75	Male	70	Temporal	ST	0	10	6.0
G87	45	Male	80	Temporal	ST	1	16	6.0
G88	71	Male	80	Parietal	ST	0	8	6.0
G89	51	Male	80	Temporal	ST	0	2	6.0
G90	57	Female	60	Parietal	ST	0	5	6.0
G91	73	Female	60	Occipital	ST	0	13	6.0
G92	54	Female	80	Parietal	T	1	15	6.0
G93#	63	Male	80	Occipital	T	0	29	6.0
G94	79	Female	80	Temporal	ST	0	9	6.0
G97	53	Male	80	Temporal	T	0	21	6.0

Surgical removal: ST- subtotal; T- total.

* Tumors analyzed by gene expression arrays.

# Only patient that remained alive at the moment of closing the study; all other patients had died.

In the present study, only patients with magnetic resonance imaging (MRI), clinical evidence of disease and a histologically confirmed diagnosis of GBM based on the World Health Organization (WHO) criteria [28], were considered.. Other criteria used for patient inclusion in the study were: i) availability of enough highly-infiltrative (>75%) tumor tissue for genetic studies, and; ii) informed consent to participate in the study given by the patient. The study was approved by the University Hospital of Coimbra Ethics Committee, according to the Declaration of Helsinki protocol.

In addition to a tumor tissue specimen, paired peripheral blood (PB) samples were also collected from each patient at diagnosis.

For every tumor sample, representative parts of fresh tumor tissues obtained by surgical resection were immediately (<30 min) snap-frozen in liquid nitrogen and stored at −80°C until used for interphase fluorescence in situ hybridization (iFISH), GEP and single-nucleotide polymorphism (SNP)-array studies. Prior to these studies, a section cut from the tissue block was assessed by conventional histopathological procedures, to estimate its tumor cell contents. Specimens with ≥75% tumor cells, in the absence of significant contamination by normal brain parenchyma and tumor necrosis, were selected for further DNA and RNA extraction, as well as for iFISH studies.

### Identification of CN alterations by SNP-arrays

For the investigation of CN alterations by SNP-arrays, DNA from frozen tumor tissue and their paired fresh PB samples was purified using the QIAamp DNA Mini Kit (Qiagen, Valencia, CA, USA) according to the instructions of the manufacturer. DNA yield and purity were determined with a NanoDrop-1000 spectrophotometer (Nano-Drop Technologies Inc., Wilmington, DE, USA) and DNA integrity was evaluated by conventional electrophoretic procedures in a 1% agarose gel. Briefly, total DNA was digested with restriction enzymes and ligated to the corresponding adaptors, following conventional Affymetrix procedures (Affymetrix Inc., Santa Clara, CA, USA). A generic primer was used in triplicate to amplify adaptor-ligated DNA fragments, through a polymerase chain reaction (PCR). After hybridization with the sample DNA, the chips were washed, labeled with streptavidin-phycoerythrin and scanned using a GeneChip Scanner 3000 (Affymetrix Inc.).

Two different SNP-array chips were used for CN analysis: 1) the GeneChip Human Mapping 500K Array Set, which provides information according to NCBI/hg17 assembly about >500,000 SNPs (262,264 SNPs in the Nsp array and 238,304 SNPs in the Sty array), was applied for the study of 23 GBM, and; 2) the Genome-Wide Human SNP Array 6.0, which contains probes for 906,600 SNPs and 945,826 non-polymorphic probes featuring a total of >1.8 million probes (Affymetrix Inc.), was used in the other 23 GBM (Table 1). Data about a total of 500,568 and 906,600 DNA probes was obtained in duplicate for paired tumor and normal PB DNA samples for each array, and it was analyzed with the Console Genotyping software (version 3.0.2; Affymetrix Inc.). In addition, the dChip 2010 software (http://www.dchip.org; Dana Farber Institute, Harvard, MA, USA) was used to calculate CN values and plot them according to chromosomal localization. Only common SNP probes between the two types of SNP-arrays (n = 481,622) were used in the analysis. Cut-off values ≤1.30 and ≥2.50 (arbitrary units) observed in tumor samples versus those obtained in the paired normal PB DNA samples, were used to establish CN losses and gains, respectively. Amplification and homozygous deletions were defined based on CN cut-off values obtained for DNA tumoral tissue of >5 and <0.8 PB DNA copies (arbitrary units), respectively.

### Gene Expression Profiles (GEP)

In a subgroup of 23 tumors, total RNA was isolated from freshly-frozen tumor tissue samples in two steps, using TRIzol (Invitrogen Life Technologies, Carlsbad, CA, USA) and the RNeasy Mini Kit (QIAGEN). The integrity and purity of the extracted RNA were determined using a microfluidic electrophoretic system (Agilent 2100 Bioanalyzer, Agilent Technologies, Palo Alto, CA, USA) and GEP were analyzed with the Gene Chip Human Genome U133 Plus 2.0 Array (Affymetrix Inc.) according to the instructions of the manufacturer, through the one-cycle cDNA synthesis kit and the Poly-A RNA gene chip control kit (Affymetrix Inc), as previously reported [29]. Datafiles containing expression values were normalized ―Robust Multi-array average expression measure (RMA)― and analyzed using the R (version 2.7.1; http://www.r-project.org) and Bioconductor software tools (http://www.bioconductor.org).

### iFISH studies

Confirmatory iFISH studies were performed in every case according to previously described methods [30], using a set of commercial dual-color fluorescence labelled probes obtained from Vysis, Inc. (Downers Grove, IL, USA) and Q-BIOgene (Carlsbad, CA, USA), as previously described in detail [29], [30]; these probes included sequences for the *TP73, ANGPTL1, EGFR, ELN, TES, p16, ABL1, PTEN, RB1, TP53, ZNF44, GLTSCR1*, and *BCR* genes (Supplementary table S2). An Axioscope fluorescence microscope equipped with a ×100 oil objective (Zeiss, Göttingen, Germany) was used to count the number of hybridization spots per nuclei (≥200 nuclei/slide). Only those spots with a similar size, intensity and shape in non-overlapping nuclei, were counted; doublet signals were considered as single spots. Briefly, gains and losses of specific chromosomal regions were considered to occur when ≥5% and ≥10% of the nuclei showed an increased and decreased number of fluorescent signals (spots) with respect to normal diploid cells, respectively. Specimens were considered to carry amplification of the *EGFR* gene when >10% of the tumor cells exhibited either an EGFR:CEP7 ratio >2 or multiple tight clusters of hybridization signals for the *EGFR* gene probe. Homozygous deletion was defined as ≥5% of tumor nuclei with centromeric probe signals in the absence of signals for the locus specific probe.

### Real Time RT-PCR validation of microarray-based mRNA levels

Microarray mRNA expression levels of four relevant genes (*BCAS2, FIP1L1, EGFR*, and *XIST*) coded in 4 different chromosomes (chromosomes 1, 4, 7 and X) were validated by an independent Quantitative Real-Time RT-PCR assay in a total of 14 GBM samples (G8, G10, G14, G17, G30,G34, G37, G40, G42, G44, G45, G46, G52 and G53). For this purpose the SuperScript III first-strand Synthesis System from Invitrogen (www.invitrogen.com) and both the LightCycler carousel-based system and the LightCycler TaqMan Master chemistry (Roche,Mannhein, Germany) were used, according to the instructions of the manufacturers.

### Statistical analyses

The statistical significance of differences observed between groups was assessed by the Student T and the Mann-Whitney U tests, for parametric and non-parametric (continuous) variables, respectively; for qualitative variables, the *X^2^* test was used (SPSS software, SPSS 15.0, SPSS Inc, Chicago, IL, USA). Survival curves were plotted according to the method of Kaplan and Meier, and the log-rank test was used to assess the statistical significance of differences in overall survival between groups of patients (SPSS software). *P*-values<0.05 were considered to be associated with statistical significance.

In order to investigate the impact of CN alterations on GEP, the relationship between DNA CN values and GEP mRNA levels was assessed for 12,445 genes investigated in common with both the SNP-arrays and the GEP arrays, using the Pearson correlation (Supplementary table S1). **The correlation between real Time RT-PCR and microarray-based mRNA levels of the** *BCAS2, FIP1L1, EGFR*, and *XIST* **genes was assessed by the Spearman correlation.**

## Results

### Chromosomal localization of amplicons and homozygous deletions

Overall, a higher number of amplicons than homozygous deletions was observed with both the 6.0 and 500KSNP-arrays. Recurrent amplicons were localized in chromosomes 7 (50% of the cases), 12 (22%), 1 (11%), 4 (9%), 11 (4%), and 17 (4%); in turn, homozygous deletions frequently involved chromosomes 9 (52%) and 10 (22%) and less frequently, chromosomes 1, 6, 12, 13, 16 and 17 (one case each).

**Figure 1:**
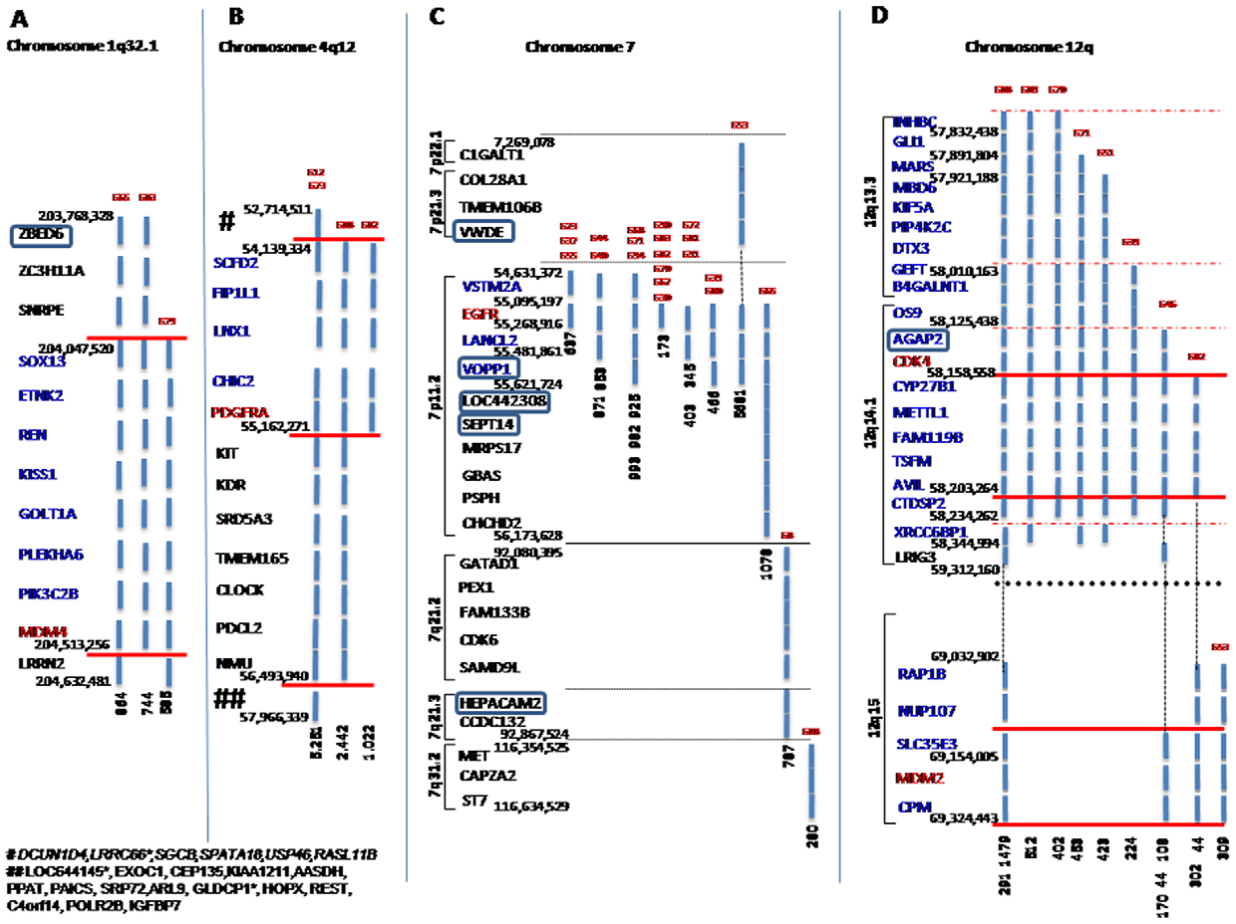
Frequently amplified chromosomal regions in GBM. Detailed characterization of the extension and the gene coded in those segments of chromosomes 1 (A), 4 (B), 7 (C) and 12 (D) found to be recurrently amplified in GBM by SNP-arrays. The identification code for each tumor isplaced on top of each line (G–N.), the length size of the amplicon in Kb is placed at the bottom of the amplified regions, and both the starting and ending positions of the amplicons are shown at the left of each chromosomal region. All genes affected in common for each amplified chromosomal segment are displayed; previously reported candidate genes amplified in a significant number of cases are shown in red, other frequently amplified genes are depicted in blue, whereas genes depicted in black correspond to genes amplified at low frequencies. A total of 6 amplified genes (DCUN1D4, LRRC66*, SGCB, SPATA18, USP46, RASL11B) and fifteen amplicons (LOC644145*, EXOC1, CEP135, KIAA1211, AASDH, PPAT, PAICS, SRP72, ARL9, GLDCP1*, HOPX, REST, C4orf14, POLR2B, IGFBP7) were additionally found in cases G12 and G73, respectively. Genes without expression values in the GEP-array are highlighted with square boxes in the figure and with an asterisk in this legend.

By far, chromosome 7p11.2 was the most frequently amplified chromosomal region (n = 21/46 patients; 46%); two additional cases showed amplicons at chromosome 7q (Figure 1 and Supplementary Table S3). In turn, a high frequency of amplicons was also noted in the long arm of chromosome 12 with recurrent involvement of the 12q14.1 (8/46 cases; 17%), 12q13.3 (6/46 patients, also showing 12q14.1 amplicons; 13%) and 12q15 (4/46 patients, three of which also showed 12q14.1 amplicons; 9%) cytobands; another four cytobands of chromosome 12 ―12q13.12, 12q13.13, 12q14.3 and 12q21.1― were affected in only one tumor each. Other recurrent amplicons involved chromosomes 4q12 (4/46 patients; 9%) and 1q32.1 (3/46 patients; 7%) (Figure 1 and Supplementary Table S3). Four additional cytobands were amplified in chromosomes 11 (11p13,11p15.3, 11q13.3 and 11q25), and 17 (17q25.1, 17q11.1, 17q11.2 and 17q24.1) in only two tumors each (Supplementary Table S3).

In some patients, coexistence of two or three amplicons in different chromosomes was observed. These included amplicons at chromosomes 4, 7 and 12 in one case (case G82), at chromosome 7 and chromosome 12 in four patients (cases G39, G53, G70 and G71), at chromosomes 4 and 12 in another two tumors (cases G82 and G88), at chromosomes 1 and 7 in two cases (cases G65 and G83), at chromosomes 7 and 11 in one patient (G23) and at chromosomes 7 and 17 in another case (G81) (Supplementary Table S3). In other cases, amplicons were restricted to a single chromosome: chromosome1 in one patient (case G79; 2%), chromosome 4 in two tumors (G12 and G73; 4%), chromosome 12 in two cases (G46 and G51; 4%) and chromosome 7 in 11 patients (G30, G37, G40, G44, G55, G67, G68, G72, G80, G91 and G94; 24%).

**Figure 2:**
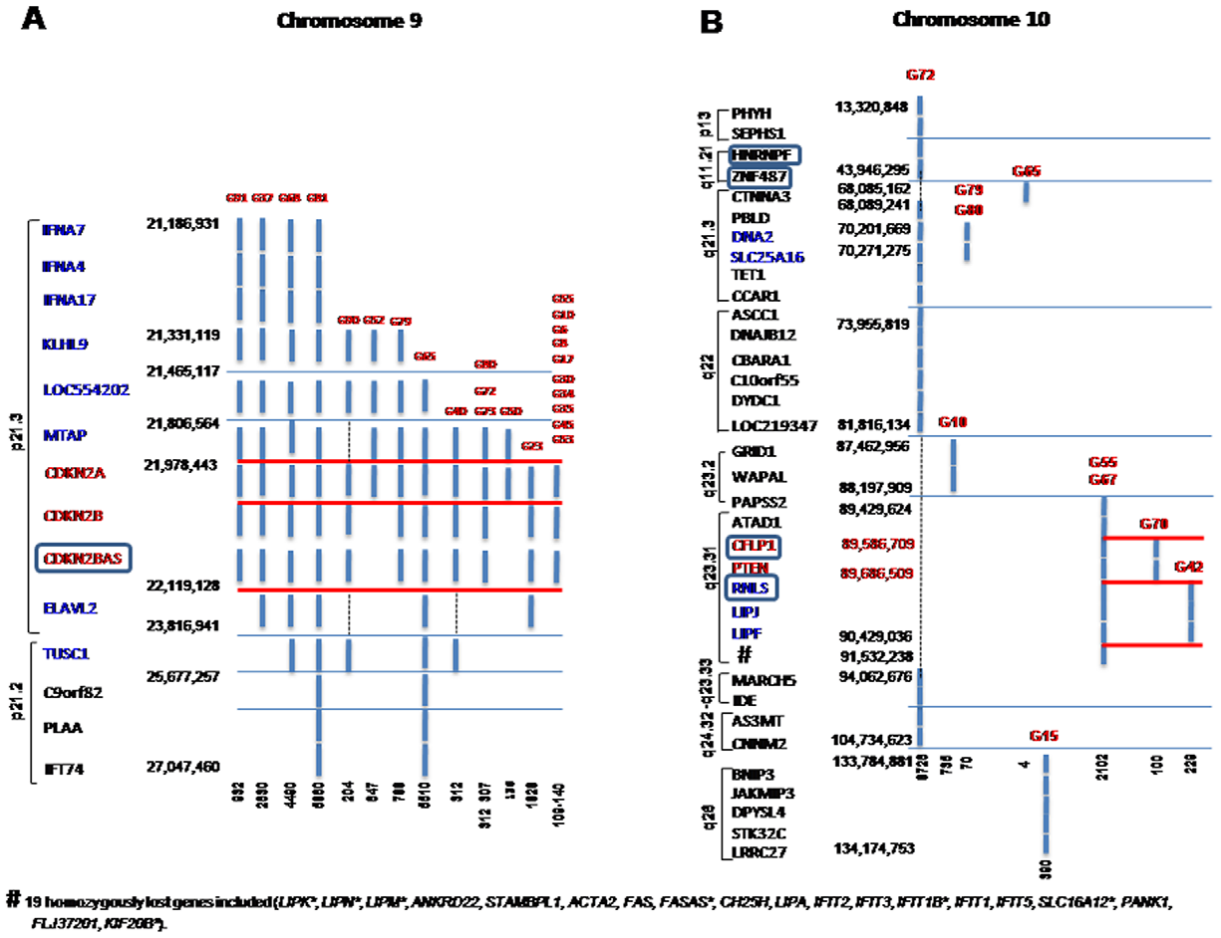
Frequently homozygously deleted chromosomal regions in GBM. Recurrent homozygously deleted segments of chromosomes 9 (9p21.2 and 9p21.3) (A) and 10 (10p13, 10q11, 10q21, 10q22, 10q23, 10q24 and 10q26) (B). The identification code for each tumor is placed on top of each line (G–N.), the length of the deleted chromosomal region in Kb is placed at the bottom of the lines corresponding to each deleted region, and both the starting and ending positions of the deleted segments are shown at the left of each chromosomal region. All genes coded in each deleted chromosomal region are displayed: previously reported candidate genes deleted in a significant number of cases are shown in red, other recurrently deleted genes are depicted in blue, while genes deleted at low frequencies are shown in black.. Genes without expression values in the array are highlighted with square boxes in the figure.

Recurrent homozygous deletions were only found for chromosomes 9 (52%) and 10 (22%) (Figure 2 and Supplementary Table S4). Despite homozygous deletion of chromosome 9 showed a highly variable extension, it mainly involved chromosome 9p21.3 and less frequently, chromosome 9p21.2, 9p22.1 and 9p23 (Supplementary Table S4). Ten tumors (G10, G15, G42, G55, G65, G67, G70, G72, G79 and G80) displayed homozygous deletions of chromosome 10 at different regions: 10q23.31, 10q23.2, 10q21.3, 10q26.3, 10p13, 10q11.21, 10q22, 10q23.33 and 10q24.32 (Figure 2 and Supplementary Table S4); homozygous deletions involving the 10q21.3 chromosomal region occurred in only four tumors (G65, G72, G79 and G80), but they frequently involved different loci; another 5 tumors (G10, G42, G55, G67 and G70) showed homozygous losses at 10q23.2–10q23.31 but, once again, they frequently involved distinct loci. Other homozygous deletions were observed in a single tumor and they involved chromosomes 13q14.2 and 12q24 (case G97), 16q22.1–16q23.2 and 1p36 (case G72), 17p12 (case G89) and 6q21 (case G80) (Supplementary Table S4).

iFISH analysis systematically confirmed the presence of the above described amplicons and homozygously deleted chromosomal regions (Supplementary Table S2); although variable patterns of numerical changes were revealed by iFISH at the single cell level, in line with previous observations [31]; such intratumoral iFISH heterogeneity was not detected by the SNP CN profiles as exemplified in Supplementary Table S5 for chromosomes 7 and 9.

### Detailed characterization of the amplified and homozygously deleted chromosomal segments

Characterization of the amplified chromosomal regions in GBM revealed variable lengths for recurrent amplicons. Overall, amplified segments in chromosome 7 ranged from 280 Mb to 5,681 Mb and those at chromosome 7p11.2 systematically included the *EGFR* gene (n = 21 samples; 46%) frequently in association with the *LANCL2* (n = 12; 26%), *VSTM2A* (n = 8;17%) and *VOPP1*(n = 7; 15%) genes (Figure 1 and Supplementary Table S3).

Recurrently amplified segments for chromosome 12 ranged from 44 Mb to 1,479 Mb and they included distinct segments localized at 12q including up to 41 different genes (Supplementary Table S3). Among other, these included the *CYP27B1*, *METTL1*, *FAM119B*, *TSFM* and *AVIL* genes at chromosome 12q14.1 in 8/46 patients (17%), and the *CDK4* and *AGAP2* genes in 7 GBM (15%). In addition, amplification of the 12q13.3 chromosomal region involved the *GEFT* and *B4GALNT1* genes in 6 patients (13%) and recurrent amplification of chromosome 12q15 involved the *MDM2* gene together with the *SLC35E3* and *CPM* genes in 4 cases (9%).

For those four tumors with amplicons at chromosome 4q12 (G12, G73, G82 and G88), the *PDGFRA, CHIC2, LNX1, FIP1L1*, and *SCFD2* genes were found to be systematically amplified, the length of the amplified chromosome 4q12 region varying between 1,022 Kb to 5,251 Kb (Figure 1).

The amplified chromosomal region at 1q32.1 (n = 3/46 tumors; 7%) showed a length of between 585 Kb and 864 Kb, and it systematically included the *MDM4* gene together with another seven genes (*SOX13*, *ETNK2*, *REN*, *KISS1*, *GOLT1A*, *PLEKHA6* and *PIK3C2B* (Figure 1 and Supplementary Table S3).

Another 10 chromosomal regions from chromosomes 1, 11 and 17 were amplified in only one tumor each and they involved a variable number of genes (between 1 and 37 genes) (Supplementary Table S3).

The most frequent homozygous deletion found involved chromosome 9p21.3 from the 21,978,443 bp to the 22,119,128 bp position. This segment included the *CDKN2A* gene in 24 patients (52%), together with the *CDKN2B* and *CDKN2BAS* genes in 22 of them (48%). The *MTAP* (12 cases; 26%), *LOC554202* (8 tumors; 17%), *ELAVL2* (5 GBM; 11%), and *TUSC1* (5 cases; 11%) genes were also frequently lost. Noteworthy, in a subset of 7 of the former 24 cases, homozygous deletion of the 9p21.3 segment extended telomericly to the 21,186,931 bp position including between three (n = 4 cases) and four (n = 3) genes more (*IFNA7, IFNA4* and *IFNA17* without or with the *KLHL9* gene, respectively) (Figure 2 and Supplementary Table S4). By contrast, the localization of the deleted regions in chromosome 10 was highly heterogeneous extending from the 13,320,848 bp to the 134,174,753 bp positions, with an overall length of between 4to 8,726 Mbp; among these cases, the *PTEN* gene was homozygously deleted in 3 tumors (Figure 2 and Supplementary Table S4). Another two genes ―*DNA2* and *SLC25A16* ―were lost at the 10q21.3 region in another 3 cases, and twenty genes coded at the 10q23.31 chromosomal region or near it ―the *ATAD1, LIPK, LIPN, LIPM, ANKRD22, STAMBPL1*, *ACTA2, FAS, FASAS, CH25H, LIPA, IFIT2, IFIT3, IFIT1B, IFIT1, IFIT5, SLC16A12, PANK1, FLJ37201* and *KIF20B* genes― were homozygously lost in 2 other cases (Supplementary Table S4). Of note, the *RB1* tumor suppressor gene coded at chromosome 13q14.2 was homozygous deleted together with the *RCBTB2* gene in only one tumor (G97) from our series (Supplementary Table S4).

### Copy number alterations and gene expression levels

Out of 12,445 genes present in common in both the SNP and GEP arrays (including 259/305 amplified or homozygously deleted genes), 46 genes showed a high correlation (R^2^>0.70) between DNA CN values and GEP RNA levels in paired GBM tumor-PB samples (n = 23) (Table 2). Noteworthy, all except three of these 46 genes were coded in those chromosomes carrying amplicons: 10 in chromosome 1, 10 in chromosome 4, 4 in chromosome 7, 1 in chromosome 11, 18 in chromosome 12. The *MTAP* gene was the only gene coded in a homozygously deleted chromosomal region (chromosome 9p21) for which a significant correlation was found between CN alterations and gene expression levels (Table 2). Of note, a high correlation was observed between the microarray expression levels and quantitative RT-PCR analysis of the mRNA levels of 4 selected relevant genes (*BCAS2, FIP1L1, EGFR* and *XIST*) with Spearman correlation coefficients of 0.6, 0.8, 0.8 and 0.9, respectively.

**Table 2:** Relationship between the CN alterations and gene expression levels for 12,445 genes analyzed in parallel with the SNP and GEP arrays, in GBM (n = 23).

Gene Name	Symbol	Cytoband	R^2^
Cold shock domain containing E1, RNA-binding	CSDE1	1p22	0.93
Transcription termination factor, RNA polymerase II	TTF2	1p22	0.75
Breast carcinoma amplified sequence 2	BCAS2	1p21-p13.3	0.90
Amylase, alpha 2B (pancreatic)	AMY2B	1p21	0.71
TryptophanyltRNA synthetase 2, mitochondrial	WARS2	1p13.3-p13.1	0.85
Synaptotagmin VI	SYT6	1p13.2	0.78
Mannosidase, alpha, class 1A, member 2	MAN1A2	1p13	0.89
Adenosine monophosphate deaminase 1 (isoform M)	AMPD1	1p13	0.83
Immunoglobulin superfamily, member 3	IGSF3	1p13	0.76
Zinc fingerprotein 697	ZNF697	1p12	0.82
Signal recognition particle 72kDa	SRP72	4q11	0.77
Transmembrane protein 165	TMEM165	4q12	0.92
FIP1 like 1 (S. cerevisiae)	FIP1L1	4q12	0.92
Exocyst complex component 1	EXOC1	4q12	0.90
Clock homolog (mouse)	CLOCK	4q12	0.88
Polymerase (RNA) II (DNA directed) polypeptide B	POLR2B	4q12	0.87
Steroid 5 alpha-reductase 3	SRD5A3	4q12	0.77
DCN1, defective in cullin neddylation 1, domain containing 4	DCUN1D4	4q12	0.75
Chromosome 4 open reading frame 14	C4orf14	4q12	0.74
Sec1 family domain containing 2	SCFD2	4q12	0.71
Transmembrane protein 106B	TMEM106B	7p21.3	0.84
Epidermal growth factor receptor	EGFR	7p12	0.85
Peroxisome biogenesis factor 1	PEX1	7q21.2	0.79
GATA zinc finger domain containing 1	GATAD1	7q21–q22	0.78
Methylthioadenosine phosphorylase	MTAP	9p21	0.73
Eukaryotic translation initiation factor 3, subunit M	EIF3M	11p13	0.78
IMP1 inner mitochondrial membrane peptidase-like *	IMMP1L	11p13	0.75
Amplified in osteosarcoma	OS9	12q13	0.86
Methyltransferase like 1	METTL1	12q13	0.82
Solute carrier family 16, member 7	SLC16A7	12q13	0.78
Cytochrome P450, family 27, subfamily B, polypeptide 1	CYP27B1	12q13.1–q13.3	0.87
Phosphatidylinositol-5-phosphate 4-kinase, type II, gamma	PIP4K2C	12q13.3	0.90
Deltex 3 homolog (Drosophila)	DTX3	12q13.3	0.75
Beta-1,4-N-acetyl-galactosaminyl transferase 1	B4GALNT1	12q13.3	0.72
Methyl-CpG binding domain protein 6	MBD6	12q13.3	0.75
Tstranslation elongation factor, mitochondrial	TSFM	12q13–q14	0.87
Carboxy-terminal domain small phosphatase 2	CTDSP2	12q13–q15	0.91
RAP1B, member of RAS oncogene family	RAP1B	12q14	0.97
Cyclin-dependent kinase 4	CDK4	12q14	0.81
Family with sequence similarity 119, member B	FAM119B	12q14.1	0.80
Advillin	AVIL	12q14.1	0.78
Glutamate receptor interacting protein 1	GRIP1	12q14.3	0.95
Mdm2, transformed 3T3 cell double minute 2	MDM2	12q14.3–q15	0.96
Nucleo porin 107kDa	NUP107	12q15	0.85
Solute carrier family 35, member E3	SLC35E3	12q15	0.79
X (inactive)-specific transcript *	XIST	Xq13.2	0.96

Only those genes (n = 46) which showed a high degree of correlation between CN alterations and RNA levels (R^2^>0.70; p-value<0.0000005) are shown.

* The *IMMP1L* (11p13) and the *XIST* (Xq13.2) genes were not amplified or deleted.

Those genes whose expression was mostly impacted by the most frequent CN alterations were the *RAP1B, TSFM, CYP27B1, METTL1, AVIL, CDK4* and *FAM119B* genes in chromosome 12q14, the *EGFR* gene in chromosome 7p11 and the *TMEM165, FIP1L1, CLOCK, SRD5A3* and *SCFD2* genes in chromosome 4q. As mentioned above the *MTAP* gene was the only gene whose expression was highly correlated to the occurrence of homozygous deletion of chromosome 9p21.

**Figure 3:**
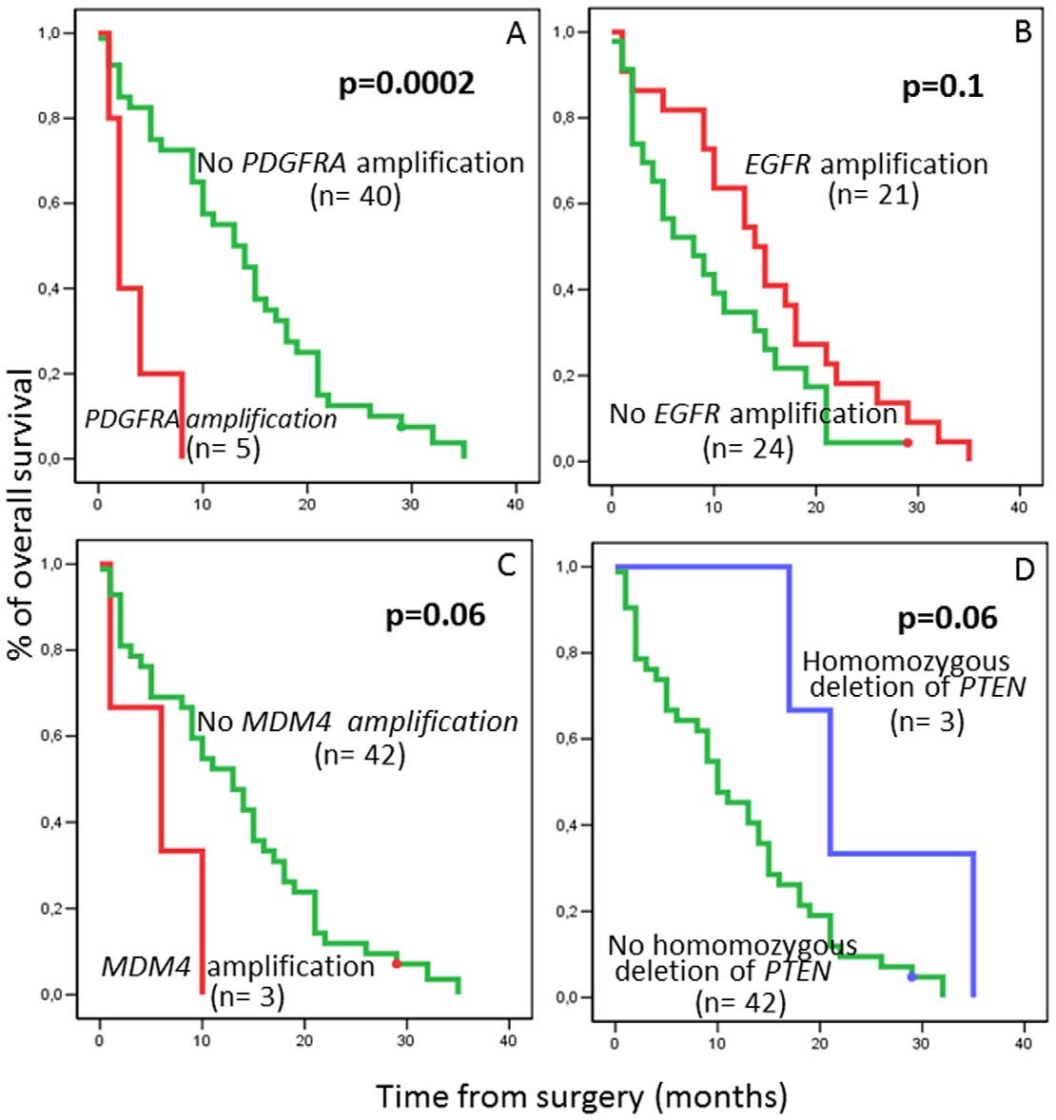
Overall survival curves of GBM patients (n = 45) according to the presence vs absence of amplification of the *PDGFRA* (Panel A), EGFR (Panel B) and *MDM4* genes (Panel C) and the presence vs absence of deletion of the *PTEN* gene (Panel D).

From the prognostic point of view, none of the amplified and homozygous deletions showed a clear impact on patient survival, except for the amplification of the *PDGFRA* gene in chromosome 4q, which was associated with a significantly shorter overall survival (median overall survival of 2 vs 13 months for the *PDGFRA* amplified vs non-amplified cases ; p = 0.0002) (Figure 3).

## Discussion

Identification of genetic markers involved in the oncogenic mechanisms and molecular pathways driving GBM, still remains a challenge. Among other approaches, detailed characterization of amplicons and homozygous deletions provides a useful tool for the screening and identification of candidate genes, despite pathogenic mechanisms (e.g. gene mutation, epigenetic silencing through gene methylation and altered expression microRNA) exist which are not directly related to CN alterations. Several techniques have been previously used for the identification of CN alterations in GBM, including high-resolution SNP-arrays [23], [32], [33], [34]. Through such approaches, CN gains of chromosome 7p11.2, together with del(9p21) and monosomy 10/del(10q), have been commonly observed in GBM, together with other less frequent abnormalities [10], [12], [35]. Although the information from such analyses is useful for the identification of both the altered chromosomal regions and the genes they contain, valuable insights into the role of relevant involved genes requires further assessment of the impact of genomic aberrations on gene expression. In this study, we integrated genomic and transcriptional data from paired DNA and RNA samples from the same tumors, in order to identify those genes involved in amplicons and homozygously deleted chromosomal regions which show a parallel change in their mRNA expression levels.

Analysis of whole-genome CN alterations by SNP-arrays confirmed the presence of previously reported recurrent genomic alterations in GBM [2], [3], [5], which were further validated here by iFISH. Overall, almost every tumor in our cohort showed CN alterations in multiple chromosomes, but their frequency and extent varied significantly among different tumors, confirming the genetic complexity of GBM [31]. Interestingly however, those regions recurrently affected by CN alterations consistent with gene amplification, were restricted to a few chromosomes (chromosomes 1q, 4q, 7p and 12q). Similarly, recurrent homozygous deletions only involved chromosomes 9p21 and 10q23. Since many genes involved in gliomagenesis often reside within amplicons and/or homozygously deleted chromosomal regions, we searched for candidate genes encoded in these regions for which a correlation existed between the DNA CN values and mRNA expression levels in the same tumor sample. Noteworthy, virtually all genes which showed a high correlation (R^2^>0.70) between DNA CN values and mRNA levels corresponded to genes coded in those six chromosomes where amplicons were recurrently observed. Amplified genes included several genes (*MDM4, PDGFRA, EGFR*, *CDK4*, and *MDM2*) whose amplification has been previously associated with the pathogenesis of GBM [8], [18], [36], [37]; in addition, other candidate genes were also found to be amplified at both the DNA and RNA levels and thus, to be potentially relevant in the pathogenesis of GBM.

In detail, the *EGFR* oncogene whose amplification defines a subset of GBM [3], [38], [39], was the only gene coded in the 7p11.2 amplicon for which a significantly high correlation between DNA CN values and gene expression levels was detected; although this amplicon contained other bystander genes that may be co-amplified with *EGFR* (e.g. *LANCL2* and *GASP*) due to their genomic proximity [14], amplification of all such genes showed limited impact on gene expression levels. These results support and reinforce the critical role of *EGFR* in the pathogenesis of a significant fraction of GBM, through activation of the RAS pathway [40], [41].

Amplification of chromosome 12q13–15 was also found in a significant proportion of primary GBM, in line with previous reports [17]. In contrast to 7p11.2 amplicons, 12q13–15 amplicons typically involved multiple genes whose expression was increased at the RNA level in parallel to the greater DNA CN values.. However, from all these genes listed in table 2, only *AVIL, FAM119B, METTL1, CYP27B1* and *TSFM* were systematically amplified in tumors displaying 12q amplicons..Co-amplification of the *CDK4* and *MDM2* genes at the 12q13–15 amplicon is frequently observed in GBM and it has been previously suggested to confer a tumor growth advantage [42]. However, in our series this amplicon showed no impact on the mRNA gene expression levels of these two genes. By contrast, relatively little is known about the role of other co-amplified and overexpressed genes which are coded in chromosome 12q13–15, even if several of them are also amplified in other tumors, e.g. in lung cancer [43]. From these genes, special attention should be paid to the *AVIL* and *CYP27B1* genes. *AVIL* encodes for advillin, a member of the gelsolin/villin family of actin regulatory proteins which is almost exclusively expressed by peripheral sensory neurons, and that has been recently identified as a new candidate driver gene in GBM [44], [45]. In turn, CYP27B1 (P450 Cytochrome 25-Hydroxyvitamin D3 1,α-Hydroxylase) catalyzes the conversion of calcidiol to calcitriol, the most active vitamin D metabolite, involved in cell proliferation with both anti-proliferative and cell differentiating effects [46], [47], [48]. Conversely, the role of *FAM119B* (a gene of unknown function, which has been associated to multiple sclerosis) [49], METLL1 ―a nuclear protein that catalyzes the formation of N(7)-methylguanine at position 46 (m7G46) in tRNA, inactivated in response to agonists of the PI3-kinase pathway or the classical MAP kinase cascade―, and *TSFM* (a gene that encodes the mitochondrial translation elongation factor EFTs) in GBM, deserves further investigations.

Amplification of chromosome 4q12 has been previously described to involve the *PDGFRA* gene in 8–15% of GBM [21], [50], as also found here. However, CN alterations at 4q12 did not show a high correlation with PDGFRA mRNA levels from the same tumor. Conversely, expression of other amplified genes coded at 4q12 in the vicinity of the *PDGFRA*gene (e.g. *FIP1L1* and *SCFD2*), appeared to be significantly modulated by the 4q12 amplicon, suggesting their potential relevance in GBM. In this regard, it should be noted that the FIP1L1 protein coded by the *FIP1L1* gene functions as a component of the cleavage and polyadenylation specificity factor (CPSF), which participates in processing of mRNA. Although, it has been observed that fusion of the *FIP1L1* gene to the *PDGFRA* gene generated by interstitial 4q12 deletion, results in a constitutively activated FIP1L1-PDGFRA fusion protein with tyrosine kinase activity in chronic eosinophilic leukemiar [51] the role of the *FIP1L1* gene in gliomagenesis, remains to be dilucidated.

Other potentially relevant genes coded at chromosome 4q12 whose CN alterations mostly impacted on their mRNA expression levels included the *TMEM165* and the *CLOCK*genes. *TMEM165* (also named *TPARL*), encodes a putative transmembrane 324 amino acid protein whose cellular functions are unknown, although intronic splice mutations of the protein have been related to congenital disorders of glycosylation [52]. Of note,*TMEM165* has been found to be up-regulated in invasive GBM cells with transcriptional differences between these and the other core tumor cells, supporting a role for this gene in tumor invasion [53]. In turn, theCLOCK protein contributes to activate transcription of the Period (PER*1*, PER*2*, and PER*3*) and Cryptochrome (CRY*1* and CRY*2*) proteins two proteins that are involved in the circadian system. Deregulation of the circadian clock protein has been implicated in many types of cancer, in both animal and humans [54], [55], [56], [57]. In a recent study on gliomas including GBM, tumor cell expression of PER1 and PER2 was significantly lower than in the surrounding normal/reactive cells [58], suggesting that deregulated expression of these two genes may result in disruption of the control of the normal circadian rhythm and a stimulatory effect on survival and proliferation of gliomas cells. However, the molecular mechanisms of genes controlling circadian rhythm in glioma cells have not been explored so far. Independently of the precise impact of the molecular mechanisms involved, amplification at the 4q12 chromosomal region was associated with a very short patient overall survival.

Amplification of other chromosomal regions such as chromosome 1q32 was found in a relatively limited number of cases, in line with previous findings [3], [8], [24]., Controversial results exist as regards the impact of 1q32 amplicon on *MDM4* and *CNTN2*, as independent versus combined targets for amplification [20]. Our results support an independent and more relevant role for the former gene as the target of 1q32 amplification, since a higher frequency of *MDM4* amplification in the absence of involvement of *CNTN2* was observed in our patients; despite this, in our series, 7 other candidate genes were amplified together with *MDM4*. These included the *PIK3C2B* gene involved in the PI3K/AKT signaling pathway, which was also amplified and overexpressed in other series of GBM [20], and the *SOX13* gene, which has been reported to be up-regulated in oligodendrogliomas [59]. Despite this, none of the amplified genes at chromosome 1q32 was associated with simultaneously increased RNA expression, further investigations being required to confirm their relevance in the pathogenesis of GBM.

Commonly deleted segments at chromosome 9p21, almost systematically involve the *CDKN2A* tumor suppressor gene, in association with both the *CDKN2B* gene, and frequently also the *MTAP* gene [60], [61], [62], [63], [64], in line with our results. *CDKN2A/B* gene products are involved in the p53 pathway through a protein―p14^arf^― encoded by an alternate reading frame, which binds to the p53/mdm complex and inhibits mdm-mediated degradation of p53. Thus, homozygous deletion of the *CDKN2A/B* locus could affect both the Rb and p53 pathways [6], [9]. Interestingly, it has been shown that *MTAP* can be lost independently of *CDKN2A*, which suggests that loss of *MTAP* may indeed play a role in tumor biology. Noteworthy, *MTAP* was the single homozygously deleted gene at chromosome 9p21 for which a high correlation was found between DNA CN values and its mRNA expression levels. These results point out the potential relevance of *MTAP* as a tumor suppressor gene. The MTAP protein is an enzyme that plays a major role in polyamine metabolism and that is essential for salvaging both adenine and methionine. This enzyme is expressed in all normal human tissues, but MTAP protein deficiency or *MTAP* gene deletion have been previously found in several tumors, including gliomas [63]. This is particularly relevant if we consider that the deleted segments at 9p21 encompassed, not only the *CDKN2A/B* tumor suppressor genes, but also numerous other potentially relevant genes, such as the *ELAVL2* (its absence in *Drosophyla* causes multiple structural defects and hypotrophy of the CNS), *KLHL9* [encodes a substrate-specific adapter of a BCR (BTB-CUL3-RBX1) E3 ubiquitin-protein ligase complex, required for mitotic progression and cytokinesis], and *IFNA* (*IFNA7*, *IFNA4*, and *IFNA17*) genes.

Monosomy 10 in association or not with del(10q) is a frequent finding in GBM. Despite multiple chromosomal segments were homozygously deleted in a small fraction of our cases, they rarely involved the same chromosomal regions and none of the deleted genes encoded in these areas, including the *PTEN* gene, showed a high correlation between its DNA CN values and mRNA gene expression levels.

In summary, here we confirm the high frequency of gene amplification in GBM, which mainly involves the 7p11.2, 12q13–15, and to a less extent also the 4q12 and 1q32 chromosomal regions. Most interestingly, amplification of those genes coded in these chromosomal segments showed a variable impact on their mRNA levels, depending on the specifically targeted gene. Conversely, recurrent homozygous deletions were restricted to chromosome 9p21 and to multiple variable segments of chromosome 10q, the MTAP gene being the only gene whose mRNA levels were significantly affected by such homozygous deletions.

## Supporting Information

Table S1 Number of probes analyzed with the two different types of SNP-arrays (6.0 and 500K SNP-arrays) and the GEP-array (U133 Plus 2.0), grouped according to their chromosomal localization. DNA CN alterations were assessed in all GBMs samples (n = 46) while mRNA expression levels were evaluated in a subset of 23 of these tumors.
(DOC)

Table S2 Commercially available dual-color iFISH probes directed against specific gene locus used in the present study.
(DOC)

Table S3 Genes amplified in GBM tumors (n = 46).
(DOC)

Table S4 GBM (n = 46): homozygously deleted genes and their corresponding cytobands in chromosomes 1, 6, 9, 10, 12, 13, 16 and 17.
(DOC)

Table S5 Copy number (CN) alterations of chromosomes 7 and 9 as assessed by SNP-arrays versus iFISH analysis.
(DOCX)
