# Supplementary material for: Amplified and Homozygously Deleted Genes in Glioblastoma: Impact on Gene Expression Levels
Source: PLoS One. 2012 Sep 28;7(9):e46088. doi: 10.1371/journal.pone.0046088 (PMC3460955; doi:10.1371/journal.pone.0046088)
Supplement: Table S1 — Number of probes analyzed with the two different types of SNP-arrays (6.0 and 500K SNP-arrays) and the GEP-array (U133 Plus 2.0), grouped according to their chromosomal localization. DNA CN alterations were assessed in all GBMs samples (n = 46) while mRNA expression levels were evaluated in a subset of 23 of these tumors. (DOC) [file pone.0046088.s001.doc]

**Table S1.** Number of probes analyzed with the two different types of SNP-arrays (6.0 and 500K SNP-arrays) and the GEP-array (U133 Plus 2.0), ~~used to study CN alterations and GEP gene expression of GBM tumors (n=46), respectively,~~ grouped according to their chromosomal localization. DNA CN alterations were assessed in all GBMs samples (n=46) while mRNA expression levels were evaluated in a subset of 23 of these tumors.

| Chromosome | SNP-array  (n=46) | | | | Common genes  for the SNP-arrays | | | GEP-array  U133 Plus 2.0  (n=23) | Common genes in the 500K SNP and GEP arrays |
| --- | --- | --- | --- | --- | --- | --- | --- | --- | --- |
|  | 6.0*SNP-array  (n=22) | | 500K^&^ SNP-array  (n=24) | |  |  |  |  |  |
|  | Total probe set | Probe set inside gene | Total probe set | Probe set inside gene | Total probe set | Probe set inside gene | Number of genes |  |  |
| 1 | 71,312 | 29,112 | 40,194 | 22,186 | 38,660 | 16,573 | 1,450 | 4,608 | 1,290 |
| 2 | 73,937 | 28,471 | 41,362 | 22,614 | 39,926 | 15,270 | 962 | 3,328 | 844 |
| 3 | 60,685 | 25,066 | 33,767 | 20,829 | 32,610 | 12,810 | 847 | 2,792 | 768 |
| 4 | 55,995 | 19,038 | 32,301 | 16,503 | 31,163 | 10,438 | 626 | 1,966 | 536 |
| 5 | 56,416 | 19,892 | 32,033 | 16,761 | 30,953 | 10,864 | 680 | 2,255 | 606 |
| 6 | 56,271 | 21,674 | 31,439 | 15,218 | 30,282 | 11,507 | 802 | 2,620 | 723 |
| 7 | 47,056 | 21,257 | 25,763 | 15,529 | 24,788 | 11,067 | 659 | 2,401 | 574 |
| 8 | 48,608 | 18,706 | 27,433 | 15,547 | 26,459 | 9,797 | 490 | 1,710 | 433 |
| 9 | 41,442 | 17,031 | 22,841 | 11,335 | 21,968 | 8,926 | 536 | 1,884 | 479 |
| 10 | 48,195 | 20,914 | 28,485 | 14,900 | 27,408 | 11,570 | 611 | 1,926 | 526 |
| 11 | 44,539 | 18,596 | 26,245 | 14,623 | 25,195 | 10,055 | 846 | 2,602 | 714 |
| 12 | 42,555 | 17,504 | 24,926 | 11,151 | 23,982 | 9,603 | 787 | 2,452 | 703 |
| 13 | 34,283 | 11,182 | 19,175 | 7,739 | 18,442 | 5,929 | 275 | 1,031 | 238 |
| 14 | 28,065 | 10,546 | 15,711 | 6,659 | 15,108 | 5,531 | 450 | 1,540 | 386 |
| 15 | 26,074 | 12,059 | 14,317 | 7,347 | 13,782 | 6,357 | 478 | 1,538 | 424 |
| 16 | 27,716 | 12,661 | 15,274 | 7,554 | 14,707 | 6,563 | 510 | 1,875 | 459 |
| 17 | 20,658 | 10,431 | 11,255 | 6,353 | 10,800 | 5,433 | 727 | 2,604 | 649 |
| 18 | 26,529 | 9,198 | 14,873 | 5,966 | 14,405 | 5,104 | 245 | 841 | 220 |
| 19 | 11,929 | 5,851 | 6,392 | 3,621 | 6,042 | 2,782 | 763 | 2,695 | 683 |
| 20 | 22,843 | 9,371 | 12,398 | 6,370 | 11,978 | 4,902 | 416 | 1,336 | 376 |
| 21 | 12,579 | 4,454 | 7,108 | 4,084 | 6,886 | 2,415 | 163 | 595 | 144 |
| 22 | 11,537 | 5,910 | 6,167 | 4,099 | 5,876 | 3,011 | 336 | 1,124 | 297 |
| X | 36,886 | 10,874 | 10,514 | 3,974 | 10,202 | 3,029 | 409 | 1,520 | 373 |
| TOTAL | 906,110 | 359,798 | 499,973 | 260,962 | 481,622 | 189,536 | 14,068 | 47,243 | 12,445 |

* Numbers indicate the number of probe sets included in the array; the 6.0 SNP-array contains 906,110 probes plus a set of 258 probes for chromosome Y and 232 mitochondrial DNA probes, apart from a set of non-informative probes not included in the table. Those probes for chromosome Y were not used in the analysis because they are not included in the 500K arrays.

^&^ 500K-arrays contain a set of 121 probes without information about them, which have been excluded from the table.
