# Supplementary material for: Amplified and Homozygously Deleted Genes in Glioblastoma: Impact on Gene Expression Levels
Source: PLoS One. 2012 Sep 28;7(9):e46088. doi: 10.1371/journal.pone.0046088 (PMC3460955; doi:10.1371/journal.pone.0046088)
Supplement: Table S2 — Commercially available dual-color iFISH probes directed against specific gene locus used in the present study. (DOC) [file pone.0046088.s002.doc]

| **Targeted gene(s)** | **Chromosome locus** | **Commercial name** | **Source** |
| --- | --- | --- | --- |
|  |  | | |
| *TP73* & *EGFL3* | 1p36.3 | LSI 1p36/LSI 1q25 and LSI 19q13/LSI 19p13 Dual Color Probe | Vysis INC |
| *ANGPTL1* & *ABL2* | 1q25 | LSI 1p36/LSI 1q25 and LSI 19q13/LSI 19p13 Dual Color Probe | Vysis INC |
| *EGFR* | 7p12 | EGFR (7p12) / Alphasatellite 7 DNA Probe, Dual-Color, Direct-Labeled | QBIOgene |
| *ELN* & *LIMK1* | 7q11.23 | LSI Williams Syndrome Region Dual Color Probe (LSI ELN/LSI 7q31 dual-color probe) | Vysis INC |
| *TES* | 7q31 | LSI Williams Syndrome Region Dual Color Probe (LSI ELN/LSI 7q31 dual-color probe) | Vysis INC |
| *p16*=*CDKN2A*=*INK4A* | 9p21 | LSI 9p21 / CEP-9 Dual Color Probe | Vysis INC |
| *ABL1* | 9q34.1 | LSI bcr/abl ES (extra signal) Dual Colour Probe | Vysis INC |
| *PTEN* | 10q23 | LSI PTEN SpectrumOrange / CEP 10 SpectrumGreen Dual Color Probe | Vysis INC |
| *RB1* | 13q14 | LSI 13 / RB-1 SpectrumOrange Probe | Vysis INC |
| *TP53* | 17p13.1 | LSI p53 SpectrumOrange Probe | Vysis INC |
| *ZNF44* & *ZK1* & *MAN2B1* | 19p13 | LSI 1p36/LSI 1q25 and LSI 19q13/LSI 19p13 Dual Color Probe | Vysis INC |
| *GLTSCR1* & *GLTSR2* & *CRX* | 19q13.3 | LSI 1p36/LSI 1q25 and LSI 19q13/LSI 19p13 Dual Color Probe | Vysis INC |
| *BCR* | 22q11.23 | LSI bcr/abl ES (extra signal) Dual Color Probe | Vysis INC |

**Table S2.** Commercially available dual-color iFISH probes directed against specific gene locus used in the present study.
