# Supplementary material for: Amplified and Homozygously Deleted Genes in Glioblastoma: Impact on Gene Expression Levels
Source: PLoS One. 2012 Sep 28;7(9):e46088. doi: 10.1371/journal.pone.0046088 (PMC3460955; doi:10.1371/journal.pone.0046088)
Supplement: Table S3 — Genes amplified in GBM tumors (n = 46). (DOC) [file pone.0046088.s003.doc]

**Table S3.** Genes amplified in GBM tumors (n=46).

| **Common Amplified Regions** | | | **ID codes of altered tumors** |
| --- | --- | --- | --- |
| **Affected cytoband** | **Start/end bp position** | **Amplified genes** |
| 1q32.1 | 204047520/204518842 | *SOX13, ETNK2, REN, KISS1, GOLT1A, PLEKHA6, PIK3C2B, MDM4* | G65,G79,G83 |
|  | 203768328/203837577 | *ZBED6, ZC3H11A, SNRPE* | G65,G83 |
|  | 204589101/204632481 | *LRRN2* | G65,G79 |
|  | 204808170/205642790 | *NFASC, CNTN2, TMEM81, RBBP5, DSTYK, TMCC2,*  *NUAK2, KLHDC8A, LEMD1, CDK18, MFSD4, ELK4, SLC45A3* | G65 |
|  | 205762139/205906385 | *SLC41A1, PM20D1, SLC26A9* | G79 |
|  | 203315126/203698379 | *FMOD, ATP2B4* | G83 |
| 1p36.21 | 13914230/15435257 | *PDPN, PRDM2, KAZ* | G89 |
| 1p12/1p13.1/  1p13.2/ 1p21.1/1p21.2 | 101386179/120272726 | *SLC30A7, DPH5, OLFM3, HEJ1, COL11A1, AMY2B, AMY2A, SYT6, TRIM33, BCAS2, DENND2C, AMPD1, NRAS, CSDE1, SIKE1, SYCP1, TSPAN2, NGF, VANGL1, CASQ2, CD58, IGSF3, CD2, PTGFRN, CD101, TTF2, TRIM45, VTCN1, MAN1A2, FAM46C, TBX15, WARS2, HAO2, HSD3B2, HSD3B1, ZNF697, PHGDH* | G8 |
| 4q12 | 53739262/55162271 | *SCFD2, FIP1L1, LNX1, CHIC2, PDGFRA* | G12,G73, G82,G88 |
|  | 55525833/56493940 | *KIT, KDR, SRD5A3, TMEM165, CLOCK, PDCL2, NMU* | G12,G73,G88 |
|  | 52714511/57966339 | *DCUN1D4, LRRC66, SGCB, SPATA18, USP46, RASL11B, LOC644145, EXOC1, CEP135, KIAA1211, AASDH, PPAT, PAICS, SRP72, ARL9, GLDCP1, HOPX, REST, C4orf14, POLR2B, IGFBP7* | G12,G73 |
| 7p11.2 | 55095197/55268916 | *EGFR* | G23,G30,G37,G39,G40,G44,G53,G55,G65,G67,G68,G70,G71,G72,G80,G81,G82,G83, G90,G91,G94 |
|  | 55440320/ 55499058 | *LANCL2* | G39,G40,G44,G53,G65,G68,G71,G72,G80,G81,G91,G94 |
|  | 54627890/ 54631372 | *VSTM2A* | G23,G37,G40,G44,G55,G68,G71,G94 |
|  | 55540791/ 55621724 | *VOPP1* | G39,G53,G65,G68,G71,G80,G94 |
| 7p22.1 | 5155327/ 12424405 | *C1GALT1, COL28A1, TMEM106B, VWDE* | G53 |
| 7p11.2 | 55095197/56173628 | *LOC442308*, *SEPT14*, *MRPS17*, *GBAS*, *PSPH*, *CHCHD2* | G65 |
| 7q21.2-7q21.3 | 92080395/92867524 | *GATAD1, PEX1, FAM133B, CDK6, SAMD9L, HEPACAM2, CCDC132* | G8 |
| 7q31.2 | 116354525/116634529 | *MET, CAPZA2, ST7* | G88 |
| 11p13 | 31547227/34376882 | *ELP4, PAX6, RCN1, WT1, EIF3M, CCDC73, PRRG4, QSER1, HIPK3, C11orf41, C11orf91, CD59, FBXO3, LMO2, ABTB2* | G23 |
| 11p15.3/  11q13.3/q25 | 12135604/134274710 | *MICAL2, TPCN2, CCND1, NTM, OPCML, SPATA19, IGSF9B, JAM3, NCAPD3, VPS2AB, ACAD8, GLB1L3, GLB1L2, B3GAT1* | G71 |
| 12q14.1 | 58156510/58203264 | *CYP27B1, METTL1, FAM119B, TSFM, AVIL* | G39,G46,G51,G70,G71,G82,G88,G89 |
|  | 58125438/58234262 | *AGAP2, CDK4, CTDSP2* | G39,G46,G51,G70,G71,G88,G89 |
|  | 58115271/58115271 | *OS9* | G39,G51,G70,G71,G88,G89 |
|  | 58341138/58344994 | *XRCC6BP1* | G51,G71,G88,G89 |
|  | 59268129/59312160 | *LRIG3* | G46,G88 |
|  | 60086206/60173878 | *SLC16A7* | G46 |
|  | 62135592/62790448 | *FAM19A2, USP15* | G88 |
| 12q14.3 | 66744517/67064865 | *GRIP1* | G46 |
| 12q13.3 | 58009372/58023981 | *GEFT, B4GALNT1* | G39,G51,G70,G71,G88,G89 |
|  | 57921188/58000216 | *MBD6, KIF5A, PIP4K2C, DTX3* | G51,G70,G71,G88,G89 |
|  | 57891804/57905687 | *MARS* | G70,G71,G88,G89 |
|  | 57832438/57861484 | *INHBC, GLI1* | G70,G88,G89 |
|  | 57317393/57442139 | *SDR9C7, TAC3, MYO1A* | G82 |
|  | 57656705/57656705 | *R3HDM2* | G70 |
| 12q13.12/12q13.13 | 51185950/52202024 | *ATF1, TMPRSS12, SCN8A* | G82 |
| 12q21.1 | 72670725/73040555 | *TRHDE* | G88 |
| 12q15 | 69154005/69324443 | *SLC35E3, MDM2, CPM* | G46,G53,G82,G88 |
|  | 69014731/69120641 | *RAP1B, NUP107* | G53,G82,G88 |
|  | 68548594/68725582 | *IFNG, IL26, IL22, MDM1* | G82 |
| 17q25.1 | 73328989/73561319 | *GRB2, RECQL5, KIAA0195, CASKIN2, LLGL2, MYO15B* | G81 |
| 17q11.1/17q11.2/17q24.1 | 25799268/64172189 | *KSR1, LGALS9, CCDC46* | G89 |
