# Supplementary material for: Amplified and Homozygously Deleted Genes in Glioblastoma: Impact on Gene Expression Levels
Source: PLoS One. 2012 Sep 28;7(9):e46088. doi: 10.1371/journal.pone.0046088 (PMC3460955; doi:10.1371/journal.pone.0046088)
Supplement: Table S4 — GBM (n = 46): homozygously deleted genes and their corresponding cytobands in chromosomes 1, 6, 9, 10, 12, 13, 16 and 17. (DOC) [file pone.0046088.s004.doc]

**Table S4.** GBM (n=46): homozygously deleted genes and their corresponding cytobands in chromosomes 1, 6, 9, 10, 12, 13, 16 and 17.

| **Affected cytoband** | **Start/end bp position** | **Deleted genes** | **ID codes of altered tumors** |
| --- | --- | --- | --- |
| 1p36.23  1p36.22  1p36.11 | 8423510/8933242  10509703/11151314  24927001/24927625 | *RERE, ENO1*  *APITD1,TARDBP, MASP2, EXOSC10*  *C1orf130* | G72  G72  G72 |
| 6q21 | 111316619/111316619 | *RPF2* | G80 |
| 9p21.3 | 21978443/21991752 | *CDKN2A* | G10, G23, G37, G40, G50, G52, G55, G65, G68, G72, G73, G79, G80, G81, G90, G91 |
|  | 22000770/22119128 | *CDKN2BAS, CDKN2B* | G10, G23, G37, G40, G55, G65, G68, G72, G73, G79, G80, G81, G90, G91 |
|  | 21806564/ 21864552 | *MTAP* | G37, G40, G50, G52, G65, G68, G72, G73, G79, G80, G81, G91 |
|  | 21465117/ 21535799 | *LOC554202* | G37, G52, G65, G68, G79, G81, G90, G91 |
|  | 21331119/ 21331119 | *KLHL9* | G37, G52, G68, G79, G81, G90, G91 |
|  | 23691051/ 23816941 | *ELAVL2* | G23, G37, G53, G65, G68, G81 |
|  | 21186931/ 21196490 | *IFNA7, IFNA4, IFNA17* | G37, G68, G81, G91 |
|  | 20351419/ 20616255 | *MLLT3* | G90, G91 |
|  | 20666605/ 21026800 | *KIAA1797, PTPLAD2* | G91 |
| 9p21.2 | 25677257/ 25677257 | *TUSC1* | G40, G53, G65, G68, G81, G90 |
|  | 26842506/ 27047460 | *C9orf82, PLAA, IFT74* | G65, G81 |
|  | 27114726/ 28652536 | *TEK, NCRNA00032, C9orf11, MOBKL2B,*  *IFNK, C9orf72, LINGO2* | G81 |
| 9p22.1 | 19520742/ 19786217 | *SLC24A2* | G90 |
| 9p22.3 | 15175597/ 15300968 | *TTC39B* | G80 |
| 9p23 | 8320501/ 10606301 | *PTRD* | G37 |
| 10q23.31 | 89579599/89686509 | *PTEN, CFLP1* | G55, G67, G70 |
|  | 90034039/90429036 | *RNLS, LIPJ, LIPF* | G42, G55, G67 |
|  | 89515818/91532238 | *ATAD1, LIPK, LIPN, LIPM, ANKRD22, STAMBPL1, ACTA2, FAS, FASAS, CH25H, LIPA, IFIT2, IFIT3, IFIT1B, IFIT1, IFIT5, SLC16A12, PANK1, FLJ37201, KIF20B* | G55, G67 |
| 10q23.2 | 89429624/ 89493239 | *PAPSS2* | G55, G67 |
|  | 87359826/ 88254361 | *GRID1, WAPAL* | G10 |
| 10q21.3 | 70174707/70271275 | *DNA2, SLC25A16* | G72, G79, G80 |
|  | 67682468/69455252 | *CTNNA3* | G65 |
| 10q26.3 | 133784881/ 134174753 | *BNIP3, JAKMIP3, DPYSL4, STK32C, LRRC27* | G15 |
| 10p13/10q11.21  10q21.3/10q22/ 10q23.33/10q24.32 | 13320848/104834876 | *PHYH, SEPHS1, HNRNPF, ZNF487, PBLD, TET1, CAR1, ASCC1, DNAJB12, CBARA1, C10orf55, DYDC1, LOC219347, MARCH5, IDE, AS3MT, CNNM2* | G72 |
| 12q24.33 | 129556460/129885628 | *TMEM132D* | G97 |
| 13q14.2 | 49065565/ 48992810 | *RB1, RCBTB2* | G97 |
| 16q22.1/16q23.2 | 68689419/ 81251846 | *CDH3, CDH1, NFAT5, NOB1, WWP2, PKD1L2* | G72 |
| 17p12 | 15854040/15884561 | *ADORA2B, ZSWIM7* | G89 |
