# Supplementary material for: Amplified and Homozygously Deleted Genes in Glioblastoma: Impact on Gene Expression Levels
Source: PLoS One. 2012 Sep 28;7(9):e46088. doi: 10.1371/journal.pone.0046088 (PMC3460955; doi:10.1371/journal.pone.0046088)
Supplement: Table S5 — Copy number (CN) alterations of chromosomes 7 and 9 as assessed by SNP-arrays versus iFISH analysis. (DOCX) [file pone.0046088.s005.docx]

Table S5. Copy number (CN) alterations of chromosomes 7 and 9 as assessed by SNP-arrays versus iFISH analysis.

| **Tumor**  **ID** | **Chromosome 7 CN** | | | **Chromosome 9 CN** | | | | |
| --- | --- | --- | --- | --- | --- | --- | --- | --- |
|  | **Locus**  **SNP Chr 7*** | **CentromericiFISH**  **probe counts**** | | **Locus**  **SNP Chr 9*^&^** | **CentromericiFISH**  **probecounts**** | | | |
|  |  | **3n** | **>4n** |  | **0n** | **1n** | **3n** | **>4n** |
| ***G15*** | *1.6-3.4* | *3%* | *17%(4n), 12%(5n), 51%(6n); 8%(8n)* | *1.3-2.6 (diploid)* | *0%* | *28%* | *29%* | *0%* |
| ***G42*** | *1.9-2.8* | *18%* | *5%(4n); 1%(6n)* | *1.6-2.4 (diploid)* | *0%* | *6%* | *6%* | *4%(4n)* |
| ***G46*** | *1.6-3.0* | *63%* | *4%(4n); 6%(6n)* | *1.4-2.4 (diploid)* | *0%* | *2%* | *8%* | *9%(4n)* |
| ***G37R-AMP*** | *1.7-8.3* | *70%* | *5%(4n); 4%(5n)* | *1.3-2.3 (diploid)* | *87%* | *6%* | *7%* | *5%(4n)* |
| ****G70-AMP*** | *1.7-9.8* | *36%* | *2%(4n)* | *1.4-2.7 (diploid)* | *0%* | *38%* | *5%* | *1%(4n)* |
| ***G88 (A7q)*** | *1.7-7.4* | *12%* | *16%(4n); 3%(5n); 9%(6n); 1%(7n)* | *1.4-2.5 (diploid)* | *0%* | *8%* | *6%* | *27%(4n)* |
| ***G89*** | *1.6-3.1* | *25%* | *21%(4n)* | *1.4-2.6 (diploid)* | *0%* | *30%* | *5%* | *14%(4n)* |
| ***G92*** | *1.9-3.2* | *11%* | *29%(4n); 3%(5n); 8%(6n)* | *1.5-2.5 (diploid)* | *41%* | *19%* | *12%* | *2%(4n)* |
| ***G92R-AMP*** | *1.2-5.6* | *28%* | *8%(4n); 2%(5n); 8%(6n)* | *1.2-2.5 (diploid)* | *75%* | *16%* | *10%* | *3%(4n)* |
| ***G93*** | *1.6-2.8* | *10%* | *13%(4n); 2%(5n); 11%(6n); 4%(7n)* | *1.5-2.6 (diploid)* | *0%* | *19%* | *9%* | *10%(4n)* |
| ***G97*** | *1.7-3.3* | *39%* | *2%(5n)* | *1.4-2.6 (diploid)* | *--* | *43%* | *5%* | *0%(4n)* |
| ***G94-AMP*** | *1.7-6.0* | *50%* | *8%(4n)* | *1.4-2.6 (diploid)* | *77%* | *10%* | *9%* | *0%(4n)* |
| ***G39-AMP*** | *1.4-9.4* | *52%* | *10%(4n); 5%(5n)* | *1.2-2.5 (diploid)* | *0%* | *1%* | *13%* | *12%(4n)* |
| ***G51*** | *1.7-3.9* | *30%* | *25%(4n); 20%(6n)* | *1.9-3.3 (gain)* | *0%* | *7%* | *20%* | *15%(4n);18%(6n)* |
| ***G71(A7q)*** | *1.7-11.4* | *21%* | *14%(4n)* | *1.5-2.9 (gain 9p24.1-22.3)^$^* | *0%* | *20%* | *12%* | *9%(4n)* |
| ***G79*** | *1.7-4.0* | *24%* | *57%(4n)* | *0.4-2.6 (del9p)* | *97%* | *11%* | *29%* | *1%(4n)* |
| ***G73*** | *1.7-3.3* | *33%* | *0%(4n)* | *0.3-2.5 (del9p)* | *83%* | *44%* | *2%* | *0%(4n)* |
| ***G37-AMP*** | *1.7-16.2* | *76%* | *10%(4n)* | *0.3-2.6 (del9p)* | *82%* | *0%* | *6%* | *4%(4n)* |
| ***G80R-AMP*** | *1.7-8.7* | *61%* | *14%(4n)* | *1.1-2.5 (del9p)* | *82%* | *7%* | *11%* | *0%(4n)* |
| ***G10*** | *1.5-3.3* | *61%* | *0%(4n)* | *0.5-2.6 (del9p)* | *72%* | *8%* | *14%* | *0%(4n)* |
| ***G8 (A7q)*** | *1.8-10.6* | *51%* | *1%(4n)* | *1.0-2.4 (del9p)* | *70%* | *4%* | *5%* | *17%(4n)* |
| ***G34*** | *1.9-2.8* | *52%* | *5%(4n)* | *1.2-2.4 (del9p)* | *0%* | *2%* | *9%* | *21%* |
| ***G35*** | *1.8-2.7* | *63%* | *6%(4n)* | *1.3-2.4 (del9p)* | *56%* | *3%* | *30%* | *14%* |
| ***G6*** | *1.6-2.9* | *15%* | *13%(4n)* | *1.1-4.1 (del9p)* | *40%* | *10%* | *2%* | *0%(4n)* |
| ***G23-AMP*** | *1.9-13.3* | *56%* | *4%(4n)* | *0.6-3.0 (del 9p)* | *59%* | *5%* | *5%* | *2%(4n)* |
| ***G68-AMP*** | *1.8-13.0* | *29%* | *1%(4n)* | *0.6-2.8 (del9p)* | *42%* | *36%* | *4%* | *0%(4n)* |
| ***G50*** | *1.6-3.0* | *49%* | *7%(4n)* | *0.8-2.4 (del9p)* | *38%* | *5%* | *7%* | *5%(4n)* |
| ***G80-AMP*** | *1.3-9.2* | *35%* | *3%(4n)* | *0.7-2.9 (del9p)* | *35%* | *22%* | *16%* | *0%(4n)* |
| ***G90-AMP*** | *1.9-12.9* | *22%* | *21%(4n); 11%(5n)* | *0.3-2.7 (del9p)* | *34%* | *26%* | *15%* | *9%(4n)* |
| ***G45*** | *1.7-2.9* | *100%* | *0%(4n)* | *0.9-2.5 (del9p)* | *13%* | *6%* | *12%* | *5%(4n)* |
| ***G13*** | *2.0-2.9* | *18%* | *11%(4n)* | *0.9-2.4 (del9p)* | *10%* | *12%* | *14%* | *13%(4n)* |
| ***G12*** | *1.6-3.2* | *19%* | *14%(4n); 11%(6n)* | *0.9-2.6 (del9p)* | *14%* | *7%* | *16%* | *11%(4n)* |
| ***G14*** | *1.7-3.4* | *8%* | *11%(4n); 21%(6n); 6%(8n)* | *1.2-2.8 (del9p)* | *0%* | *6%* | *20%* | *9%(4n)* |
| ***G67-AMP*** | *2.2-11.3* | *21%* | *47%(4n)* | *1.0-2.6 (del9p)* | *0%* | *0%* | *21%* | *43%(4n)* |
| ***G83-AMP*** | *1.7-8.5* | *50%* | *4%(4n)* | *1.3-2.4 (del9p)* | *0%* | *18%* | *11%* | *0%(4n)* |
| ***G66*** | *1.4-3.6* | *32%* | *6%(4n)* | *1.1-2.7 (del9p)* | *0%* | *12%* | *13%* | *2%(4n)* |
| ***G52*** | *1.9-3.0* | *16%* | *13%(4n); 34%(6n)* | *0.6-2.3 (-9p)* | *51%* | *15%* | *4%* | *4%(4n)* |
| ***G53-AMP*** | *1.7-17.4* | *6%* | *15%(4n); 42%(6n)* | *0.5-2.5 (-9p)* | *6%* | *19%* | *4%* | *4%(4n)* |
| ***G55R-AMP*** | *1.4-9.8* | *13%* | *3%(4n)* | *0.6-2.5 (-9p)* | *8%* | *17%* | *16%* | *0%(4n)* |
| ***G87*** | *1.9-3.1* | *15%* | *2%(6n)* | *1.3-2.6 (-9p)* | *0%* | *26%* | *0%* | *0%(4n)* |
| ***G44-AMP*** | *1.5-18.0* | *45%* | *7%(4n);4%(5n);9%(6n)* | *0.5-2.3 (del9p & del9q)* | *0%* | *42%* | *3%* | *7%(4n)* |
| ***G91-AMP*** | *1.7-12.7* | *69%* | *0%(4n)* | *0.2-2.4 (del9p & del9q)* | *92%* | *34%* | *0%* | *0%(4n)* |
| ***G81-AMP*** | *1.5-14.6* | *20%* | *16%(4n);15%(5n);13%(6n)* | *0.5-2.6 (partial -9p & -9q)* | *100%* | *4%* | *60%* | *8%(4n)* |
| ***G72-AMP*** | *1.4-11.6* | *41%* | *9%(4n)* | *0.3-2.8 ( homo del(9p) & cnLOH9p)* | *87%* | *10%* | *41%* | *10%(4n)* |
| ***G55-AMP*** | *1.6-12.0* | *46%* | *0%(4n)* | *0.5-3.6 (cnLOH 9p)* | *28%* | *25%* | *3%* | *0%(4n)* |
| ***G30-AMP*** | *1.2-10.4* | *10%* | *0%(4n)* | *1.0-2.5 (cnLOH & del9p)* | *23%* | *4%* | *7%* | *3%(4n)* |
| ***G40-AMP*** | *1.9-8.2* | *7%* | *48%(4n)* | *0.4-2.5 (cnLOH & del9p)* | *45%* | *6%* | *10%* | *17%(4n)* |
| ***G17*** | *1.6-3.0* | *14%* | *2%(4n); 13%(6n);13%(9n)* | *1.1-2.5 (-9)* | *12%* | *23%* | *6%* | *0%(4n)* |
| ***G65-AMP*** | *2.0-10.6* | *20%* | *15%(4n); 13%(5n); 7%(6n)* | *0.4-2.2 (-9)* | *84%* | *23%* | *6%* | *2%(4n)* |
| ***G82-AMP*** | *1.7-7.3* | *19%* | *40%(4n); 20%(5n)* | *1.2-2.1 (-9)* | *0%* | *18%* | *53%* | *0%(4n)* |

*Results expressed as copy number values and ** as percentage of altered cells.
